# Artificial Legs for Civilians

**Published:** 1923-02

**Authors:** Henry H. C. Baird

**Affiliations:** Captain (Late Editor, *The Ex-Service Man*). Bridge, Canterbury


### Artificial Legs for Civilians.

Sir,—As a layman, I suppose it is altogether out of order for me to invite the hospitality of your columns on the subject of artificial legs, but as seldom a day passes without my receiving letters of inquiry from civilians who have had the misfortune to lose a leg, I thought I might, as a thoroughly contented wearer of an artificial leg (above knee amputation), be forgiven for intruding.

All I want to do is to express the hope that all interested in the supply of artificial legs, whether these be surgeons, Societies, hospitals, or Medical Officers attached to large industrial concerns, will investigate the merits of the Duralumin leg, with double swivel pelvic band, now issued to ex-Service men of all ranks by the Ministry of Pensions—or perhaps I should say to all of these who may be fortunate enough to hear of it, and then apply for it.

I make this request for two reasons. First because I am quite convinced that this type of artificial leg has only to be seen for the enormous advantages of it to be appreciated; and secondly because I, and I am sure all other ex-Service men, are naturally anxious that the civilian shall have equal opportunity with us of obtaining the very best type of leg available if for no other reason that their misfortune is identical with ours. The knowledge that they are still being dragged down both mentally and physically by the heavy obsolete type of wooden leg disturbs us.

There are, I understand, some fifteen limb-fitting centres within the British Isles where this type of Duralumin leg can be seen, and it is now available for all types of amputations; and should any of your readers wish for such further particulars as a mere layman wearer is able to give, I shall be happy to supply them.

Henry H. C. Baird,
Captain
(Late Editor, *The Ex-Service Man*).

Bridge, Canterbury.

